# Supplementary material for: Lymphocyte activation gene 3 (Lag3) expression is increased in prion infections but does not modify disease progression
Source: Sci Rep. 2018 Oct 2;8:14600. doi: 10.1038/s41598-018-32712-8 (PMC6168446; doi:10.1038/s41598-018-32712-8)

Lymphocyte activation gene 3 (Lag3) expression is increased in prion infections but does not modify disease progression

Yingjun Liu [1]*, Silvia Sorce [1], Mario Nuvolone [1, 2], Julie Domange [1], Adriano Aguzzi [1]*

1 Institute of Neuropathology, University of Zurich, Schmelzbergstrasse 12, CH-8091 Zurich, Switzerland

2 Amyloidosis Research and Treatment Center, Foundation Scientific Institute Policlinico San Matteo, Department of Molecular Medicine, University of Pavia, Pavia, Italy

*Corresponding author:

Adriano Aguzzi

Institute of Neuropathology, University of Zurich

Schmelzbergstrasse 12, CH-8091 Zurich, Switzerland

Tel: +41 44 255 2107; Fax: +41 44 255 44 02

Or

Yingjun Liu

Institute of Neuropathology, University of Zurich

Schmelzbergstrasse 12, CH-8091 Zurich, Switzerland

Tel: +41 44 255 32 36; Fax: +41 44 255 44 02

Supplementary Fig 1.

Depletion of Lag3 in the brain of Lag3 KO mice. qRT-PCR results of Lag3 expression in the brains of Lag3 WT and KO mice. *** P< 0.001 (n=3).

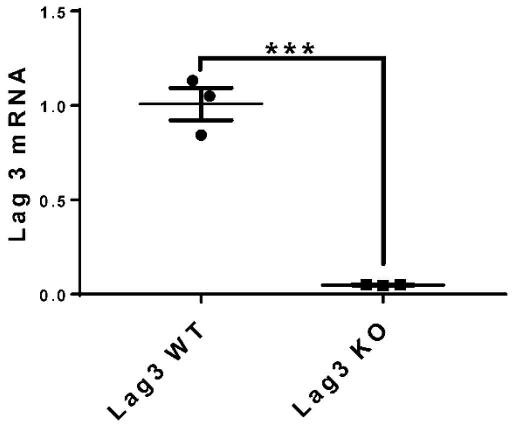

Supplementary Fig 2.

PrP^C protein levels in the brains of Lag3 WT and KO mice. A, representative images of PrP^C western blot in the brains of Lag3 WT and KO mice. **B**, quantitative data of PrP^C protein levels shown in **A**. n=5. n.s, not significant. Full-length images of the blots shown in A are included in Supplementary Fig 3.

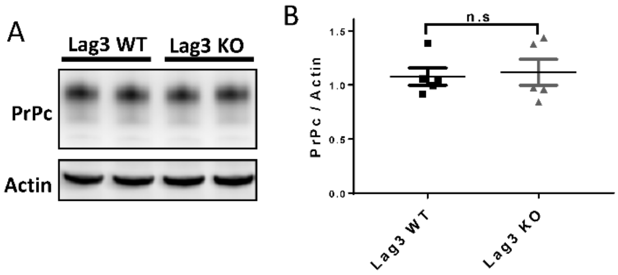

Supplementary Fig 3.

Full-length images of the blots shown in Supplementary Fig 2 A.

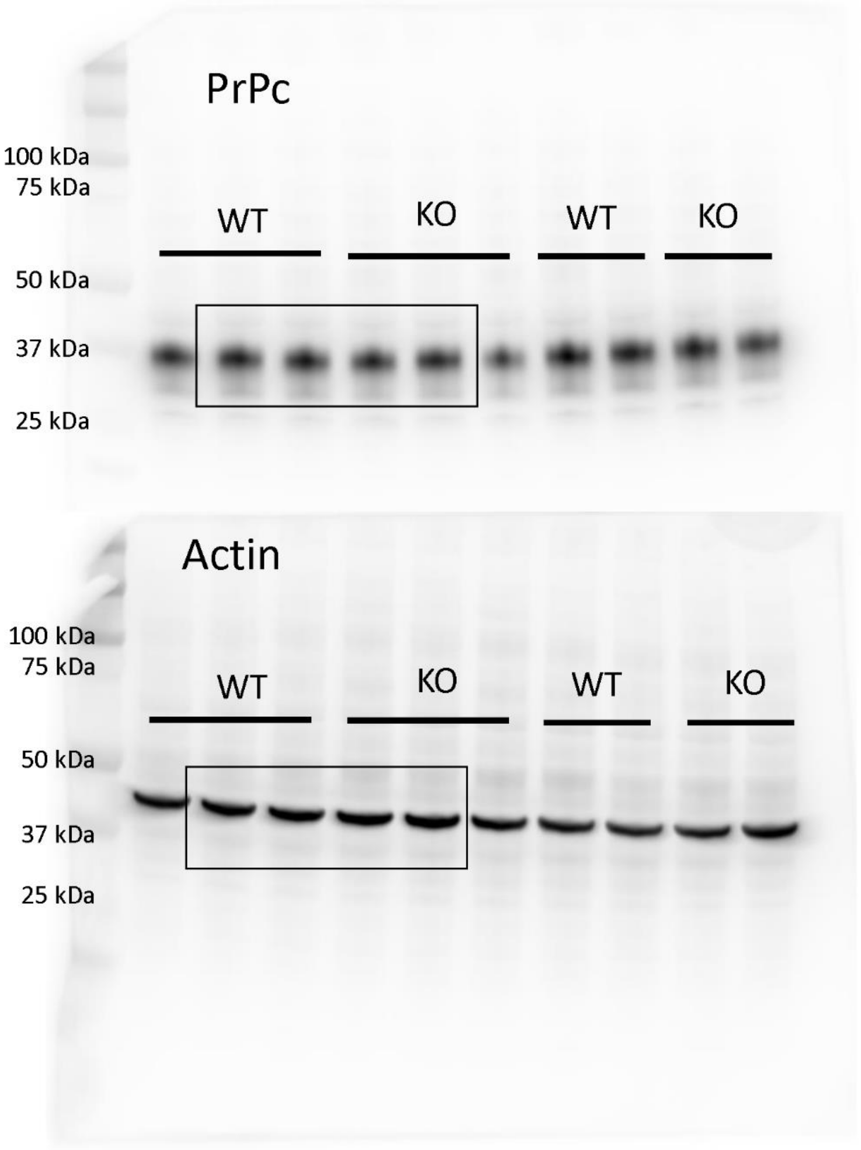

Supplementary Fig 4.

Full-length image of the blot shown in Fig 2 B.

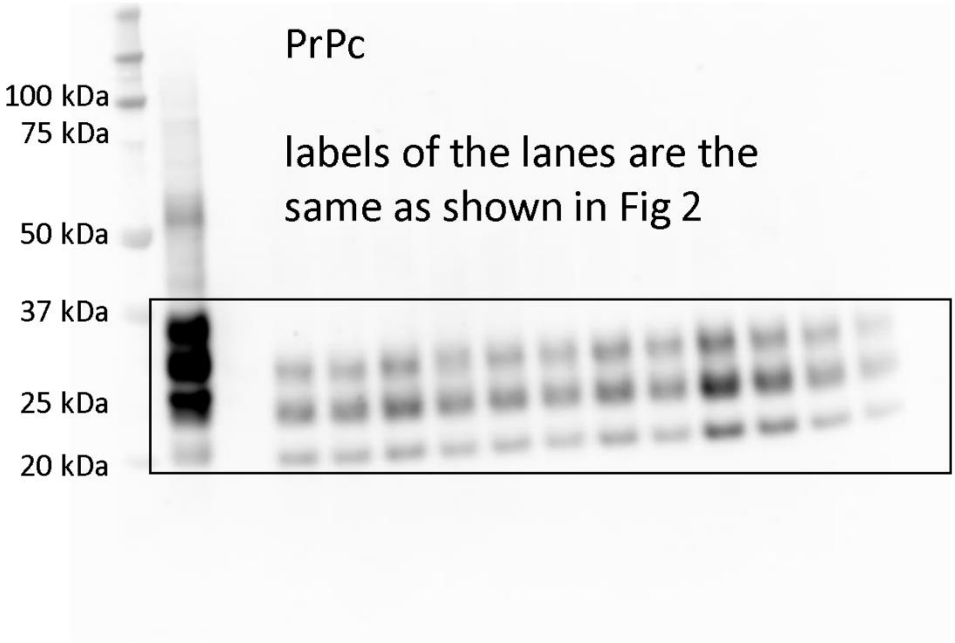

Supplementary Fig 5.

Full-length images of the blots shown in Fig 3 A.

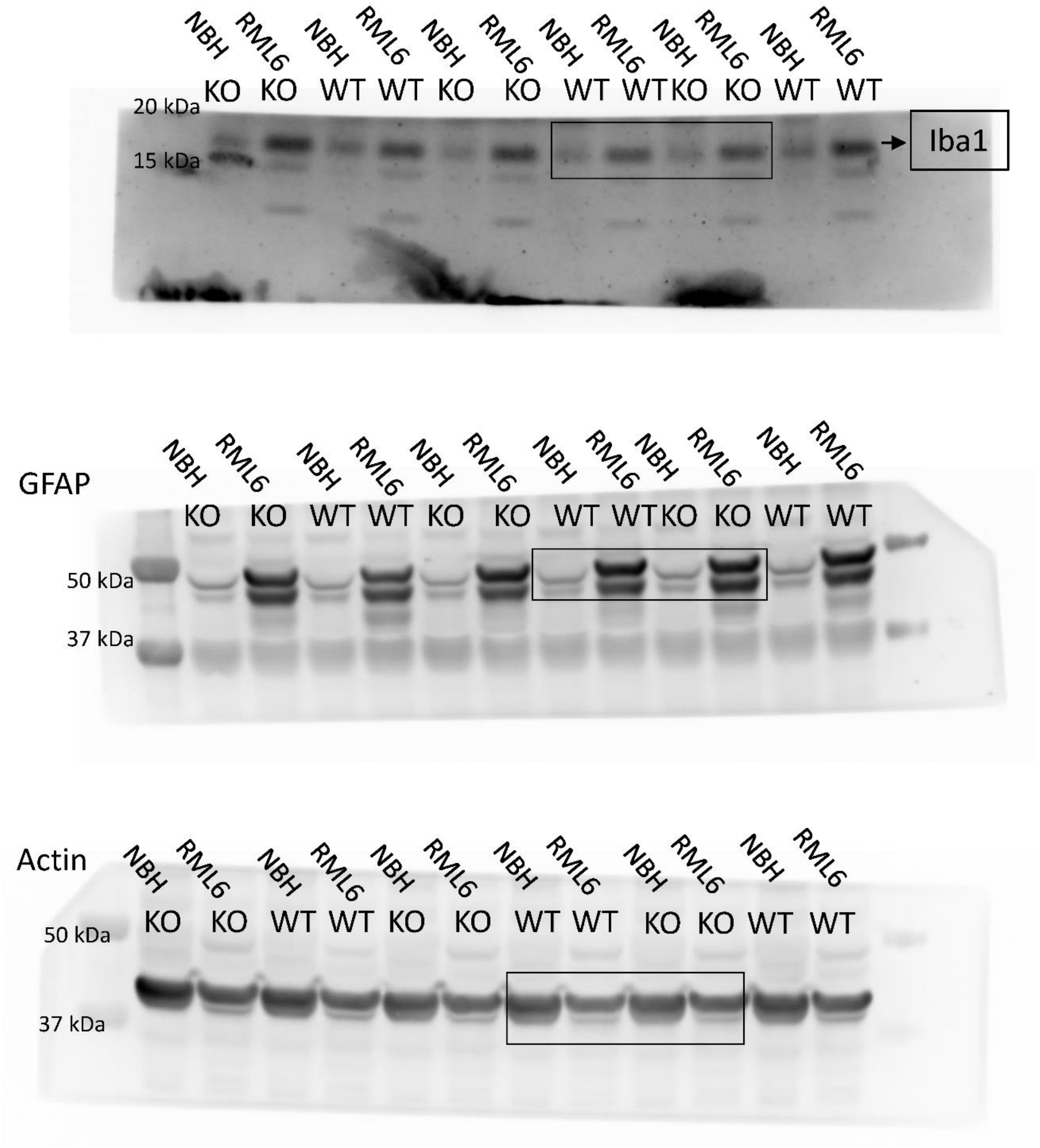

Supplement: Supplementary file 1 — Supplementary material [file 41598_2018_32712_MOESM1_ESM.pdf]
